# Geniposidic acid alleviates osteoarthritis progression through inhibiting inflammation and chondrocytes ferroptosis

**DOI:** 10.1111/jcmm.18228

**Published:** 2024-03-23

**Authors:** Jiayang Sun, Xianji Song, Cuijie Wang, Qing Ruan

**Affiliations:** ^1^ Department of Orthopedics China‐Japan Union Hospital of Jilin University Changchun Jilin China; ^2^ Department of Anesthesiology China‐Japan Union Hospital of Jilin University Changchun Jilin China

**Keywords:** chondrocytes, ferroptosis, Geniposidic acid, IL‐1β, osteoarthritis

## Abstract

Osteoarthritis is one of the common diseases that seriously affects the quality of life of middle‐aged and elderly people worldwide. Geniposidic acid (GPA) is extracted from *Eucommia ulmoides* that exhibits various pharmacological effects. This study investigated the function of GPA on osteoarthritis (OA) in IL‐1β‐stimulated mouse chondrocytes and mouse OA model. Mouse OA model was established by destabilization of the medial meniscus (DMM) and GPA was given intraperitoneal injection. The results demonstrated that GPA could alleviate DMM‐induced OA in mice. In vitro, IL‐1β‐induced PGE2, NO, MMP1 and MMP3 were suppressed by GPA. Furthermore, IL‐1β‐induced ferroptosis was inhibited by GPA, as confirmed by the inhibition of MDA, iron, and ROS, as well as the upregulation of GSH, GPX4, and Ferritin. In addition, GPA was found to increase the expression of Nrf2 and HO‐1. And the inhibition of GPA on IL‐1β‐induced inflammation and ferroptosis were prevented by Nrf2 inhibitor. In conclusion, GPA alleviates OA progression through inhibiting inflammation and chondrocytes ferroptosis via Nrf2 signalling pathway.

## 1 INTRODUCTION

Osteoarthritis (OA) is the most common chronic degenerative disease of bones and muscles worldwide and one of the main causes of disability.^1^ Its main characteristics are articular cartilage loss, synovitis changes and joint tissue remodelling.^2^ The main manifestations are periarticular pain, deformity, stability and decreased motor function, which have serious negative effects on patients' physical and mental health, quality of life and other aspects.^3^ As an age‐related joint disease, about 18% of women and 9.6% of men over 60 years old in the world have symptoms of OA, and its incidence rate is still rising.^4^ Because the pathogenesis of OA has not been fully clarified, at present, most treatment strategies for OA focus on relieving symptoms rather than reversing the development of the disease. Therefore, patients in advanced stage of OA have to accept joint replacement without better choice. At present, the injection of celecoxib into the articular cavity is a common method for the treatment of knee OA, which is conducive to the formation of a protective layer and plays the role of protecting and repairing the damaged articular cartilage.^5^ However, there are still some patients with knee OA treated with celecoxib with unsatisfactory results.^6^ In recent years, Chinese herbal medicine has played an important role in the treatment of OA and can reduce the damage of cartilage cells in OA.^7^

Ferroptosis is a novel form of iron dependent cell death that differs significantly from other types of programmed cell death in morphology, biochemistry, and genetics, such as apoptosis, necrosis and autophagy.^8^ The imbalance of iron homeostasis has adverse effects on the differentiation and function of osteoblasts and osteoclast.^9^ Iron overload will interfere with bone formation, induce bone loss, and cause osteoporosis.^10^ In the animal model of iron overload, it can be observed that a large amount of intracellular iron accumulates in the cartilage of the femoral head and the patellar fat pad of the knee joint, causing changes in iron transporters, cytokines, and cartilage structure, which is an important factor in the early occurrence of OA.^11^ The transport protein DMT1, which transports iron into cells, is an important cause of iron overload.^12^ The use of DMT1 inhibitors can reduce inflammation and ECM degradation, and delay the process of OA.^13^ Iron overload is the primary link that causes lipid peroxide aggregation and induces ferroptosis. Triggering ferroptosis can affect the pathological and physiological processes of OA.^14^

Geniposidic acid (GPA) is a bioactive compound extracted from *Eucommia ulmoides* and can suppress inflammation and oxidative stress.^15^ GPA could attenuate mouse lung injury induced by LPS via suppressing inflammation.^16^ GPA could alleviate DSS‐induced colitis through regulating gut microbiota and inflammatory response.^17^ Furthermore, GPA exhibited anti‐inflammatory roles in periodontitis induced by bacteria in mice.^18^ In addition, GPA attenuated ANIT‐induced cholestatic liver inflammation through regulating NLRP3 signalling pathway.^19^ GPA also exhibited neuroprotective roles in Alzheimer's disease (AD) mouse model.^20^ However, the roles of GPA on OA remain unclear. The purpose of this study was to explore the roles of GPA on OA and clarify the possible mechanism.

## 2 MATERIALS AND METHODS

### 2.1 Reagents

GPA (purity >98%) was purchased from MedChemExpress (NJ, USA). MMPs, NF‐κB, GPX4 and Nrf2 signalling antibodies were obtained from CST (MA, USA). Recombinant human IL‐1β and ELISA kits were obtained from R&D systems (MN, USA). Griess reagent, MDA, GPX assay kits were acquired from Nanjing Jiancheng Bioengineering Institute (Nanjing, China).

### 2.2 Cells and treatment

Under sterile conditions, the knee joints were amputated using bone scissors. Place in a sterile dish, scrape off the surrounding synovial tissue, and rinse with D‐hanks solution. The muscle cartilage on the joint surface is cut off with a surgical blade and cut into about 2 mm^3^ fragments with ophthalmic scissors. The tissues were added with 5 mL 0.25% trypsin, shaked and digested at 37°C for 30 min. Then discard the supernatant, wash it with D‐Hanks solution for three times, and add 5 mL 0.2% type II Collagenase, and continue to shake and digest it at 37°C. Cells are collected every 1 h to prevent a decrease in activity due to prolonged digestion. Transfer the cell suspension into a 15 mL centrifuge tube at 1200 *r*/min, centrifuge for 8 min, discard the supernatant, wash with D‐Hanks solution three times, and filter with 150 mesh nylon mesh into a sterile culture flask. The cells were cultured in DMEM medium containing 10% Fetal bovine serum, 100 U/mL penicillin and 100 U/mL Streptomycin.

### 2.3 CCK8 assay

Chondrocytes were seeded in 96 well plates at the density of 3 × 10^4^ pieces/mL, and the cells were treated with different concentrations of GPA, each group is set with six composite wells. After each group of cells is cultured in the corresponding culture medium for 24 h, the absorbance value at a wavelength of 450 nm is detected by an enzyme‐linked immunosorbent assay. Select three GPA concentrations with no significant cytotoxicity for subsequent experiments.

### 2.4 Measurement of MMPs, PGE2 and NO

ELISA was applied to detect the content of inflammatory mediators, including PGE2, MMP1, and MMP3 in chondrocytes of each group and OA mice according to the manufacturer's instructions. The production of NO was measured by detecting the level of accumulated nitrite in the supernatants by the Griess reagent.

### 2.5 Ferroptosis detection

Ferroptosis was measured by detecting the levels of MDA, GSH and iron concentration, as well as GPX4 expression. The concentrations of MDA, GSH and iron were tested by the assay kits according to the instructions. GPX4 expression was detected by western blot analysis.

### 2.6 Nrf2 knockdown experiment

Chondrocytes were seeded into six well plates and transfected with siRNA (Nrf2 or control) using the transfection reagents (Santa Cruz, USA). After 24 h, GPA (50 μM) was added and stimulated with IL‐1β for 24 h. Then, the production of MMPs, PGE2, and NO were measured.

### 2.7 In vivo study

Thirty‐six 8‐week‐old male C57 mice weighing (24.19 ± 1.73) g were obtained from Jilin University. The animal experiment was approved by the Ethical Committee of Jilin University. The breeding temperature is 22°C ~ 24°C, and the humidity is 30% ~ 70%. The mice were randomly divided into three groups, control group, OA group and OA + GPA (50 mg/kg) group, with 12 animals in each group. The OA model was established as previously described.^21^ The anaesthetised mice were shaved off from their knee joints and their skin disinfected. Expose the trochlear groove of the knee joint: make a smooth incision at the knee joint with a scalpel, passively divide the skin and muscle of the mouse, expose the knee joint, open the incision muscle again near the knee joint, turn the muscle to the opposite side and expose the trochlear groove of the knee joint. Expose the anterior cruciate ligament: carefully separate the joint fat pad using micro tweezers, bend the mouse knee joint to 90° and expose the anterior cruciate ligament. Verify the modelling effect through drawer experiments.

### 2.8 Western blot analysis

The cartilage tissue was ground on ice to powder, and then added to RIPA to extract total protein. BCA detection method is used for protein quantification. 30 μg of protein was separated on SDS‐PAGE and transferred it to a PVDF membrane, and sealed it with western sealing solution at room temperature for 1 h. The proteins were incubated with primary antibodies (1:500) and a 1:10000 diluted secondary antibody. Then, it was developed using a highly sensitive ECL luminescent solution. Image‐Pro Plus 6.0 software was used to measure the grayscale values of each swimlane protein, and represent its relative expression level as the target protein grayscale value/internal reference β‐actin protein grayscale value.

### 2.9 H&E staining

Cartilage tissues were fixed for 24 h and make it into 1 cm^3^ specimen. The specimen was placed in a 30% formic acid formaldehyde solution for decalcification for 1 week. After decalcification, rinse with running water for 30 min, gradient ethanol dehydration, xylene transparency and paraffin embedding, the samples were sliced into 5 μm and perform H&E staining.

### 2.10 Statistical analysis

SPSS 20.0 was used to analyse the data. Two independent sample *t*‐tests were used for mean comparison between the two groups, and one‐way ANOVA was used for mean comparison between multiple groups. When *p* < 0.05, the difference is statistically significant.

## 3 RESULTS

### 3.1 Effects of GPA on chondrocyte viability

**FIGURE 1:**
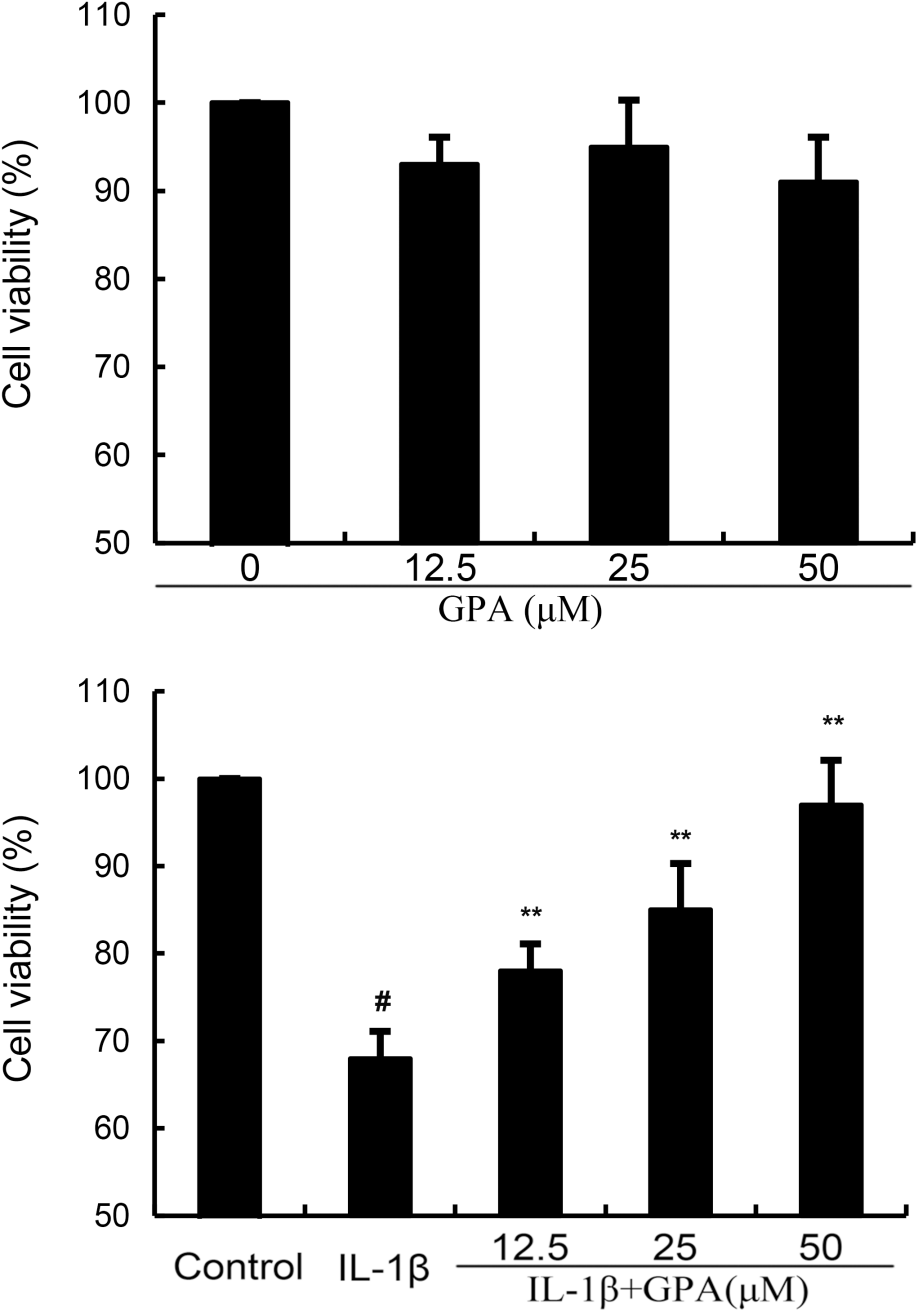
The effects of geniposidic acid (GPA) on the cell viability of osteoarthritis chondrocytes. Cells were cultured with different concentrations of GPA (12.5, 25 and 50 μM) for 24 h. The cell viability was determined by CCK8 assay. The values presented are the means ± SEM of three independent experiments. #*p* < 0.05 versus control group; **p < 0.01 versus IL‐1β group.

The role of GPA on the viability of chondrocytes was tested by CCK8 assay. GPA at the dose of 0–50 μM did not affect the viability of chondrocytes (Figure 1). Thus, 12.5, 25 and 50 μM GPA were used for the following study. Meanwhile, IL‐1β decreased the viability of chondrocytes and GPA (12.5, 25 and 50 μM) could increase the viability of chondrocytes decreased by IL‐1β (Figure 1).

### 3.2 GPA attenuates IL‐1β‐induced inflammation and MMPs production

**FIGURE 2:**
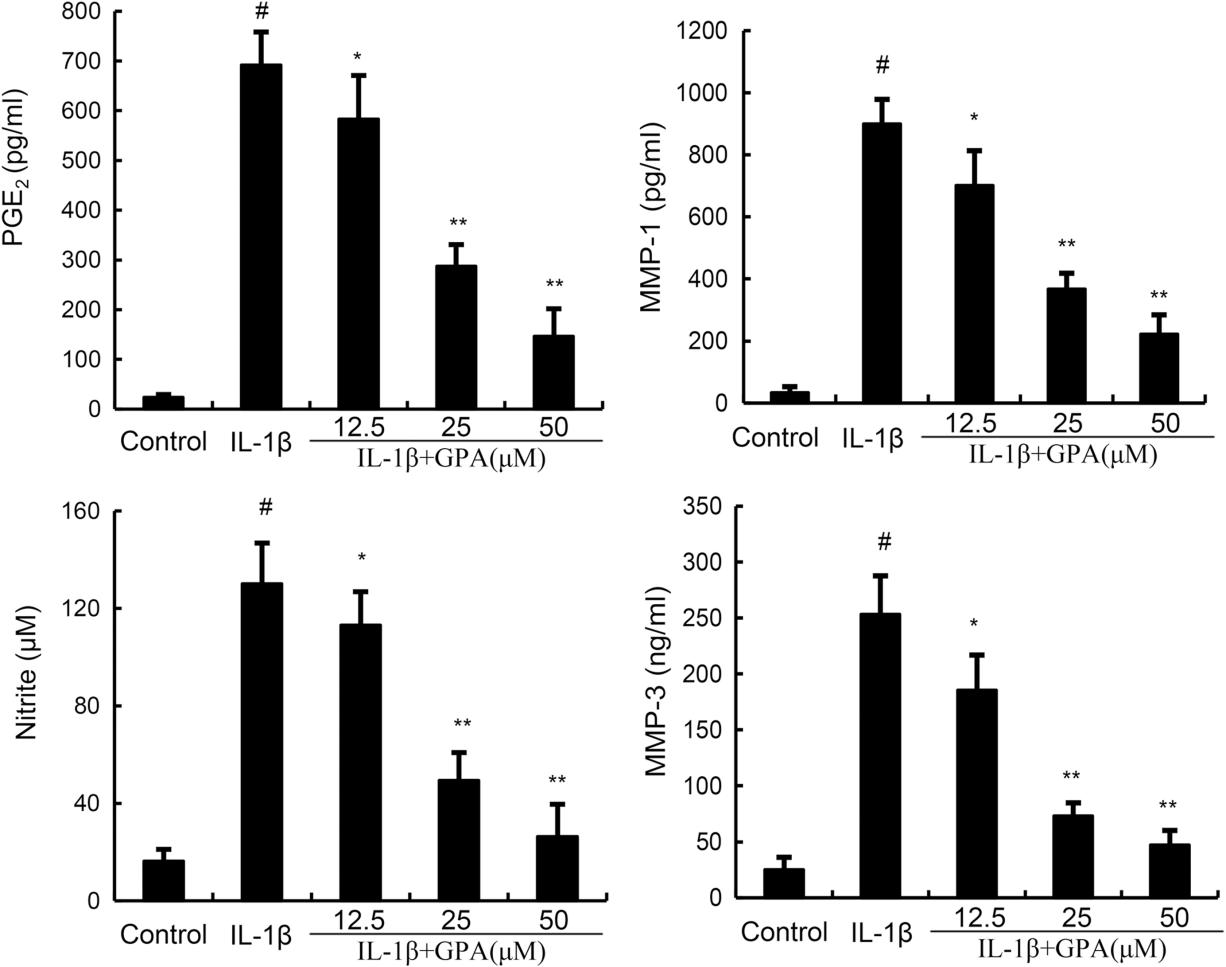
Geniposidic acid (GPA) inhibits IL‐1β‐induced PGE2, NO, MMP1 and MMP3 production. The data presented are the means ± SEM of three independent experiments. #*p* < 0.05 versus control group; **p < 0.01 versus IL‐1β group.

Inflammation and MMPs play a critical role in the pathogenesis of OA. To assess the protective effects of GPA on OA, NO, PGE2, MMP1 and MMP3 were tested in this study. As shown in Figure 2, the production of NO, PGE2, MMP1 and MMP3 were prominently increased in IL‐1β‐stimulated chondrocytes. GPA at the dose of 12.5, 25 and 50 μM markedly decreased NO, PGE2, MMP1 and MMP3 production in IL‐1β‐stimulated chondrocytes (Figure 2). These results indicated GPA could inhibited IL‐1β‐induced inflammation and MMPs production.

### 3.3 GPA alleviates chondrocyte ferroptosis induced by IL‐1β

**FIGURE 3:**
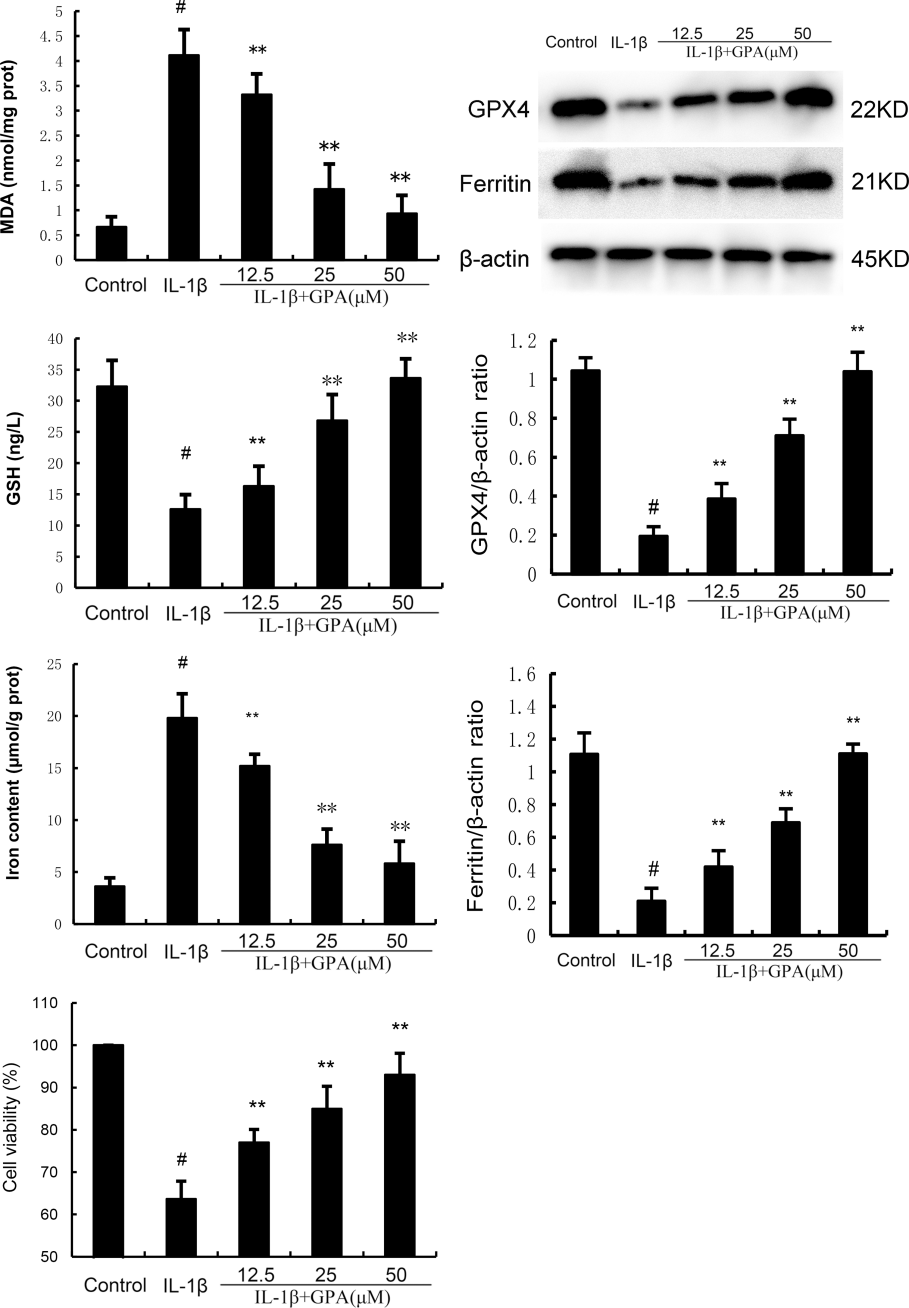
Geniposidic acid (GPA) inhibits IL‐1β‐induced ferroptosis. The values presented are the means ± SEM of three independent experiments. #*p* < 0.05 versus control group; *p < 0.05, **p < 0.01 versus IL‐1β group.

Ferroptosis also plays an important role in the pathogenesis of OA and inhibition of ferroptosis has protective role on OA. Therefore, the role of GPA on chondrocyte ferroptosis was tested in this study. As shown in Figure 3, the production of MDA and iron were prominently increased in IL‐1β‐stimulated chondrocytes. GPA at the dose of 12.5, 25 and 50 μM markedly decreased MDA and iron production in IL‐1β‐stimulated chondrocytes (Figure 3). The expression of GPX4, ferritin and GSH production were prominently decreased in IL‐1β‐stimulated chondrocytes. GPA at the dose of 12.5, 25 and 50 μM markedly increased GPX4 and ferritin expression, and GSH production in IL‐1β‐stimulated chondrocytes (Figure 3). These results suggested that GPA could inhibit IL‐1β‐induced ferroptosis. Meanwhile, the cell viability of chondrocytes was decreased by IL‐1β and GPA could increase the cell viability of chondrocytes in a concentration dependent manner (Figure 3).

### 3.4 The role of GPA on IL‐1β‐induced NF‐κB activation

**FIGURE 4:**
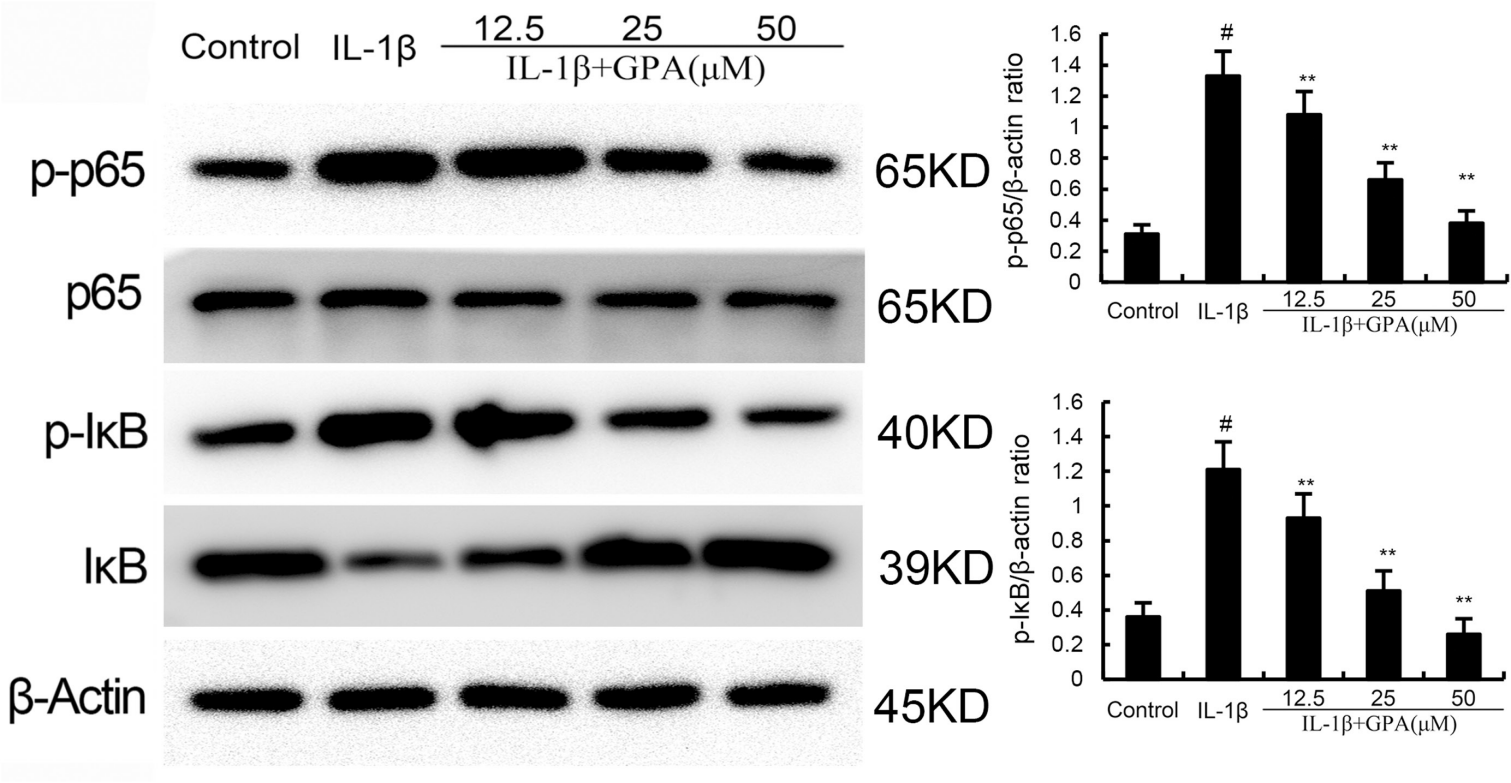
Geniposidic acid (GPA) inhibits IL‐1β‐induced NF‐κB activation. The values presented are the means ± SEM of three independent experiments. #*p* < 0.05 versus control group; *p < 0.05, **p < 0.01 versus IL‐1β group.

NF‐κB is a transcription factor that regulates the expression of various pro‐inflammatory genes. To investigate the anti‐inflammatory mechanism of GPA, NF‐κB activation was tested by western blot analysis. As shown in Figure 4, the expression of phosphorylated NF‐κB p65 and IκBα were prominently increased in IL‐1β‐stimulated chondrocytes. GPA at the dose of 12.5, 25 and 50 μM markedly decreased phosphorylated NF‐κB p65 and IκBα expression in IL‐1β‐stimulated chondrocytes (Figure 4). These results suggested that GPA could inhibit IL‐1β‐induced NF‐κB activation.

### 3.5 GPA inhibits inflammation and ferroptosis through activating Nrf2

**FIGURE 5:**
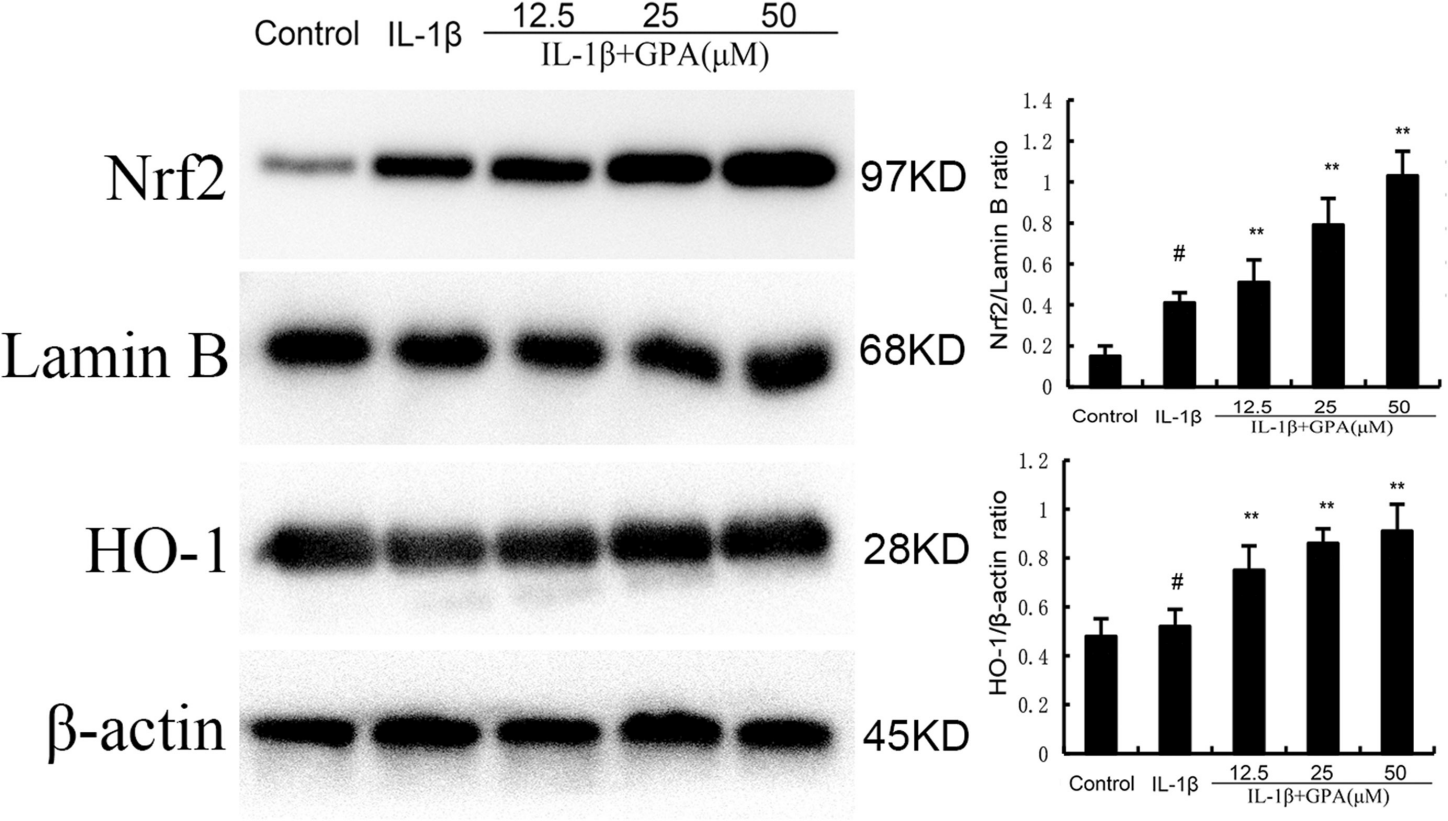
Effects of geniposidic acid (GPA) on Nrf2 and HO‐1 expression induced by IL‐1β. The values presented are the means ± SEM of three independent experiments. #*p* < 0.05 versus control group; *p < 0.05, **p < 0.01 versus IL‐1β group.

**FIGURE 6:**
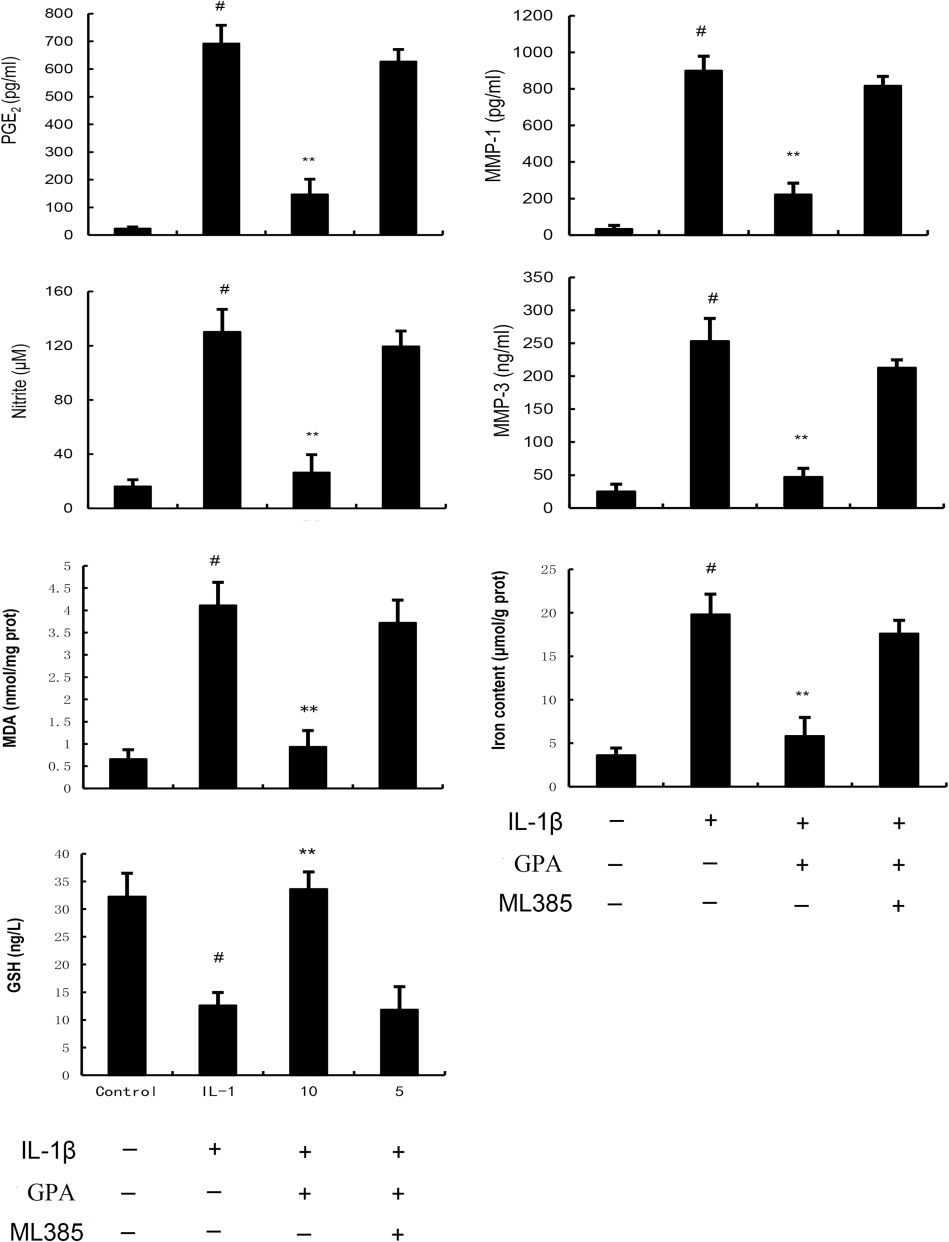
The inhibition of geniposidic acid (GPA) on IL‐1β‐induced inflammation, MMPs production and ferroptosis were prevented by Nrf2 inhibitor. The values presented are the means ± SEM of three independent experiments. #*p* < 0.05 versus control group; *p < 0.05, **p < 0.01 versus IL‐1β group.

**FIGURE 7:**
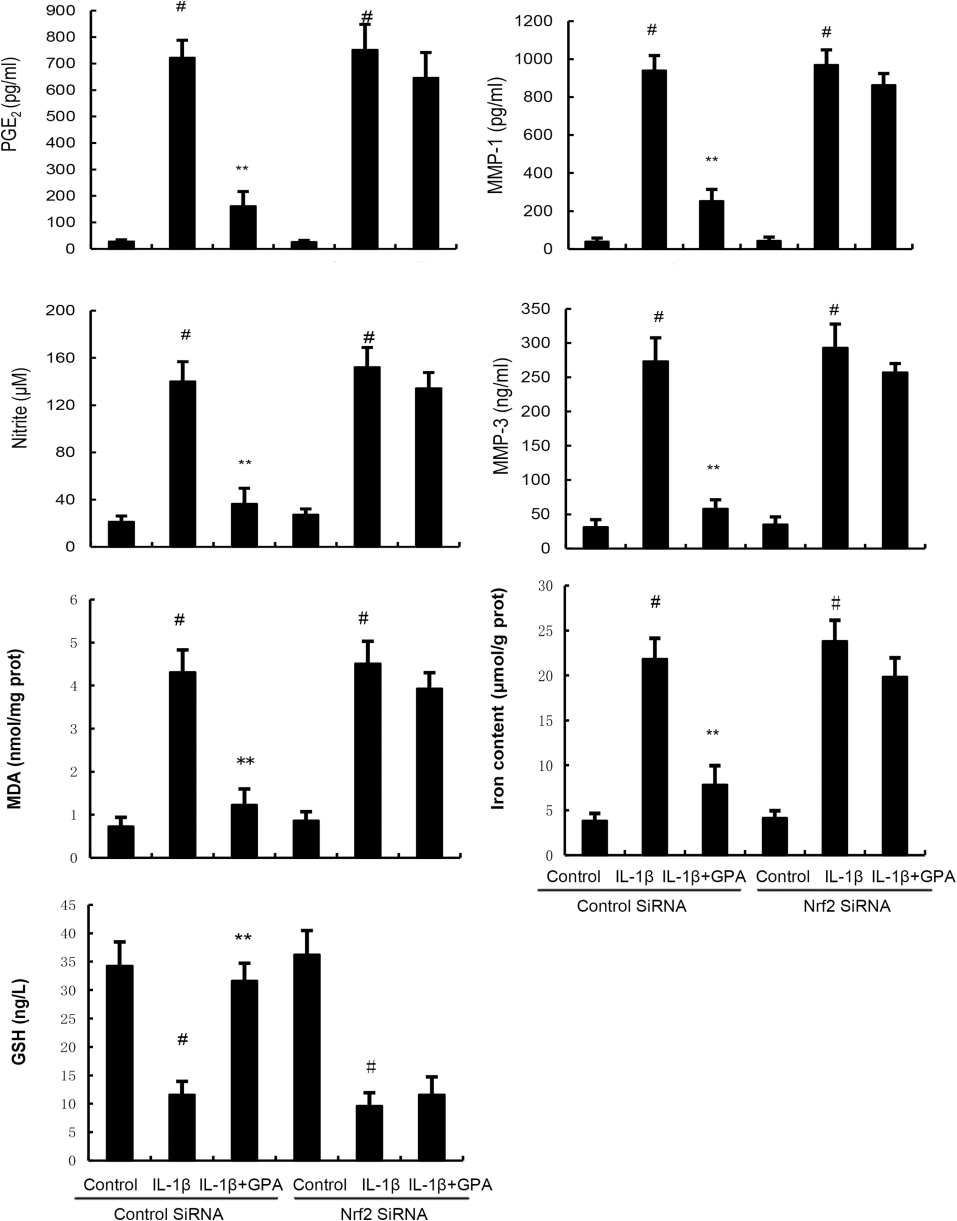
The inhibition of geniposidic acid (GPA) on IL‐1β‐induced inflammation, MMPs production and ferroptosis were prevented when Nrf2 was knockdown. The values presented are the means ± SEM of three independent experiments. #*p* < 0.05 versus control group; *p < 0.05, **p < 0.01 versus IL‐1β group.

Nrf2 is a transcriptional regulatory factor for cellular defence against oxidative stress. Activation of Nrf2 could inhibit inflammation and ferroptosis. Therefore, the effect of GPA on Nrf2 expression was tested in this study. As shown in Figure 4, the expression of Nrf2 and HO‐1 increased in IL‐1β‐stimulated chondrocytes. GPA at the dose of 12.5, 25 and 50 μM prominently increased Nrf2 and HO‐1 expression in IL‐1β‐stimulated chondrocytes (Figure 5). Furthermore, the inhibitory role of GPA on IL‐1β‐induced inflammation and ferroptosis were reversed by Nrf2 inhibitor ML385 or when Nrf2 was knockdown (Figures 6 and 7). These results suggested that GPA inhibited inflammation and ferroptosis through activating Nrf2.

### 3.6 GPA inhibits OA development in a DMM model

**FIGURE 8:**
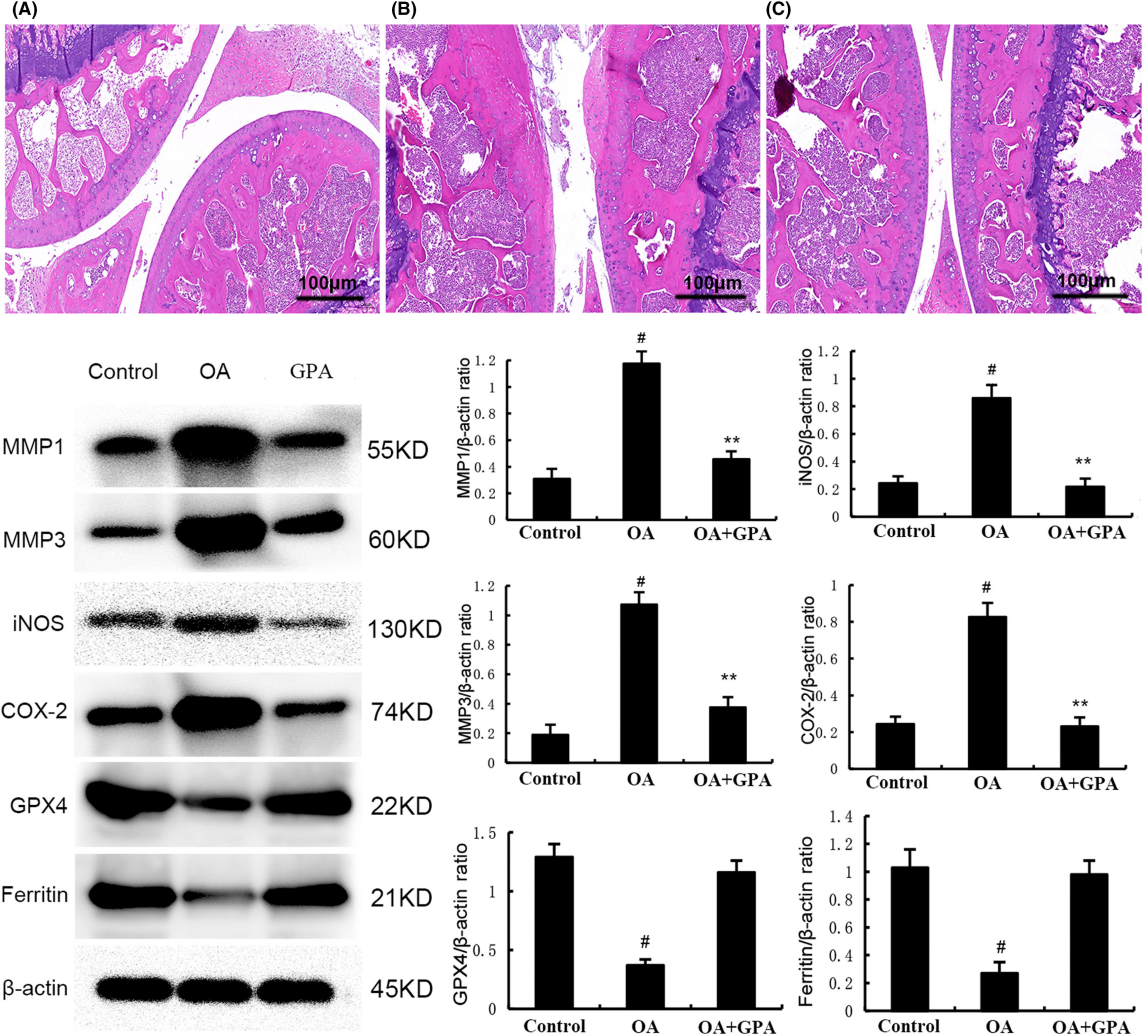
Geniposidic acid (GPA) inhibits OA development in a destabilization of the medial meniscus (DMM) model. The values presented are the means ± SEM of three independent experiments. #*p* < 0.05 versus control group; **p < 0.01 versus osteoarthritis (OA) group.

Compared with the sham surgery group, the superficial cartilage structure of the destabilization of the medial meniscus (DMM) model group was severely damaged, and obvious cartilage fibrosis was seen with the decrease of cartilage thickness. The cracks reached to the hypertrophic cartilage layer, and the entire articular cartilage presented an irregular morphological structure. On the contrary, GPA treatment can significantly increase the thickness of cartilage and reduce the number of fibrocartilage. The surface chondrocytes are regularly arranged and in good shape (Figure 8).

## 4 DISCUSSION

The most common drugs for treating OA in clinical practice are non‐steroidal anti‐inflammatory drugs (NSAIDs) and COX‐2 inhibitors, which can improve symptoms of chronic pain and joint swelling.^22^ However, long‐term use of NSAIDs and COX‐2 inhibitors can cause damage to the digestive system and kidneys, and NSAIDs cannot effectively reverse the process of cartilage degeneration.^23^ Therefore, exploring new ways to treat OA and studying safe and non‐toxic functional food ingredients and nutrients in an economical and practical manner has become crucial for alternative prevention and treatment. In this study, we found GPA had therapeutic effect against OA through inhibiting inflammation and ferroptosis.

OA is a common progressive and degenerative arthropathy in clinic.^24^ The specific mechanism of OA pathogenesis is not yet very clear, and the treatment of OA can only be limited to symptomatic management.^25^ In the past, OA was considered as a degenerative disease, and its pathological changes mainly included articular cartilage wear, subchondral osteosclerosis and periarticular osteophyte hyperplasia.^26^ However, with the deepening of research, the important role of inflammatory response in the pathogenesis of OA has gradually received widespread attention.^27^ In the OA process, pro‐inflammatory factors and inflammatory mediators released by immune cells and extracellular matrix degradation trigger the immune regulatory process.^28^ The inflammatory factors produced by stimulating the immune system include TNF‐α, IL‐1β, IL‐6 and proteases such as matrix metalloproteinases (MMP‐1, MMP‐3 and MMP‐13).^29^ These factors lead to the degradation of cartilage ECM structure, damage to subchondral bones and ligaments, and enlargement of joint cysts. Studies demonstrated that inhibition of inflammatory response could attenuate the development of OA.^30^ In this study, GPA markedly inhibited IL‐1β‐induced inflammatory mediator and MMPs production. The NF‐κB signalling pathway is closely related to the growth, survival, proliferation, apoptosis, differentiation, and inflammatory response of chondrocytes.^31^ So far, many studies have shown that pro‐inflammatory cytokines activate the NF‐κB pathway by binding to corresponding receptors, leading to a signalling cascade reaction of p65 protein phosphorylation and p65 nuclear translocation.^32^ The phosphorylation of related pathway proteins promotes the overexpression of inflammatory factors such as iNOS, COX‐2, IL‐6 and MMPs, thereby damaging articular cartilage and accelerating the process of articular cartilage degradation. Inhibition of NF‐κB activation could alleviate OA.^33^ In this study, we found GPA significantly inhibited IL‐1β‐induced NF‐κB activation.

Ferroptosis is a special form of programmed cell death that is iron ion dependent and driven by lipid peroxidation.^34^ The three basic characteristics of ferroptosis are lipid peroxide clearance dysfunction, the presence of redox active iron, and oxidation containing polyunsaturated fatty acid phospholipids. Recent studies demonstrated that ferroptosis inhibitor had protective role against OA.^35^ Ferroptosis can be used as a target for OA treatment.^36^ Glutathione peroxidase 4 (GPX4) is the main regulator of ferroptosis. Previous studies have confirmed that ferroptosis inducer erastin can promote fibroblast differentiation into myofibroblasts by increasing lipid peroxidation and inhibiting GPX4 expression.^37^ Nuclear factor E2 related factor 2 (Nrf2), as the main transcriptional regulatory factor for cellular defence against oxidative stress, is associated with transcriptional activation and ferroptosis.^38^ Chondrocyte ferroptosis plays a critical role in the pathological process of OA and activating of Nrf2 could inhibit chondrocyte ferroptosis and OA.^39^, ^40^ In this study, we found GPA markedly increased the expression of Nrf2 and HO‐1. Nrf2 inhibitor could reverse the inhibition of GPA on IL‐1β‐induced inflammation and ferroptosis, indicating GPA exhibited OA protective role through activating Nrf2 signalling.

Our results demonstrated GPA exhibited protective role in OA both in vitro and in vivo through inhibiting inflammation and ferroptosis. However, other respects, such as apoptosis, subchondral bone, involved in the pathogenesis of OA, have not been investigated. In further study, we also considered using cartilage explants for further studies.

In conclusion, GPA could alleviate OA development in mice and inhibit IL‐1β‐induced inflammation and ferroptosis through activating Nrf2/GPX4 signalling pathway. GPA can be used as an agent for the treatment of OA.

## AUTHOR CONTRIBUTIONS

**Jiayang Sun:** Data curation (equal); investigation (equal); methodology (equal); software (equal); writing – original draft (equal). **Xianji Song:** Data curation (equal); formal analysis (equal); investigation (equal); methodology (equal); resources (equal); validation (equal). **Cuijie Wang:** Data curation (equal); project administration (equal); software (equal); supervision (equal); validation (equal); visualization (equal). **Qing Ruan:** Conceptualization (equal); investigation (equal); project administration (equal); resources (equal); validation (equal); visualization (equal); writing – review and editing (equal).

## CONFLICT OF INTEREST STATEMENT

The authors have no relevant financial or non‐financial interests to disclose.

## CONSENT FOR PUBLICATION

All authors agree to publish in Journal of Cellular and Molecular Medicine.

## Data Availability

The data that support the findings of this study are available from the corresponding author upon reasonable request.
